# Variation in the androgen receptor gene exon 1 CAG repeat correlates with manifestations of autoimmunity in women with lupus

**DOI:** 10.1530/EC-14-0039

**Published:** 2014-05-05

**Authors:** Nancy J Olsen, Ann L Benko, William J Kovacs

**Affiliations:** 1 Division of Rheumatology College of Medicine, Milton S. Hershey Medical Center, The Pennsylvania State University Mail Code H044, 500 University Drive, Hershey, Pennsylvania, 17033-0850 USA; 2 Division of Endocrinology, Diabetes and Metabolism College of Medicine, Milton S. Hershey Medical Center, The Pennsylvania State University Mail Code H044, 500 University Drive, Hershey, Pennsylvania, 17033-0850 USA

**Keywords:** androgen receptor, humoral autoimmunity, lupus

## Abstract

Clinical and experimental evidence support a role for gonadal steroids in modulating the expression and course of autoimmune diseases such as lupus. Whether or not inherited variation in sensitivity to circulating androgenic hormones could influence the manifestations of such disease is, however, unknown. We sought to determine whether differences in androgen sensitivity conferred by variation in the exon 1 CAG repeat region of the androgen receptor (*AR*) gene were associated with differences in the clinical or humoral immune manifestations of lupus in a cohort of female subjects. We found that shorter *AR* CAG repeat lengths in lupus subjects correlated with a higher Systemic Lupus Erythematosus Disease Activity Index score, higher ANA levels, and expression of a broader array of IgG autoantibodies. Our findings of more severe clinical manifestations and more exuberant humoral autoimmunity in women with a shorter *AR* exon 1 CAG repeat length suggest a role for genetically determined sensitivity to androgens as a modulator of autoimmune processes.

## Introduction

Androgenic gonadal steroid hormones exert effects on both human autoimmune disease and the corresponding animal models. In an experimental mouse model of human systemic lupus erythematosus, females acquire fatal renal disease while males are generally spared; treatment of females with androgens prevents the progression of nephritis while castration of males results in disease progression and mortality (1, 2, 3). Clinical evidence in human disease also suggests that androgens can modulate autoimmunity. An association of Klinefelter's syndrome with lupus has been reported in several studies, and in isolated case reports, reversal of hypogonadism in such patients with Klinefelter's syndrome by testosterone replacement has been accompanied by evidence of remission of the autoimmune process (4, 5, 6, 7, 8). We have recently sought to investigate whether inherited differences in hormonal sensitivity might influence the expression of autoimmune processes in persons with no evidence of hormonal excess or deficiency states.

Inherited variation in the androgen receptor (*AR*) gene affects androgen action. Deleterious mutations in *AR* can result in syndromes ranging from mild abnormalities to total failure of normal male phenotypic development (9, 10). AR is a ligand-activated transcription factor with a domain structure that includes ligand-binding, DNA-binding, and transcriptional activation domains – all of which are required for the full activity of the protein. Exon 1 of the normal *AR* gene also includes a CAG repeat region of variable length (usually between 15 and 30 repeats) that encodes a polyglutamate tract in the N-terminal region of the protein (11). Kennedy's disease, a form of spinal and bulbar muscular atrophy accompanied by insensitivity to androgen signaling, results from massive expansion of the number of CAG repeats in this region (12). However, even within the range of variation observed among normal individuals, the length of this repeat region (and the encoded Glu_n_ sequence in the protein) is inversely related to the capacity of the receptor to activate a target gene *in vitro* (13, 14, 15, 16). *AR* signaling capacity *in vitro* diminishes by an average of 1.7% for each additional CAG repeat within the range from 16 to 35 (17). In humans, such variation in *AR* CAG repeat lengths has been found to be associated with phenotypic features of men with Klinefelter's syndrome (18), as well as with normal variation in facial and body hair (19), in body composition (20), in HDL levels (21), and in response to treatment with exogenous androgens (22). In a previous study, we found that longer *AR* CAG repeats were associated with more exuberant expression of IgG autoantibodies in males with lupus (23), while two recent reports have now identified inverse correlations between *AR* CAG repeat length and disease activity in women with rheumatoid arthritis (24) and lupus (25).

We reasoned that, as women have much lower circulating levels of endogenous androgens, inherited variation in sensitivity to these hormones might play an even more significant role in modulation of immune reactivity when compared with men. We genotyped a cohort of women with lupus for variation at the *AR* exon 1 CAG repeat and correlated the genotypes with clinical manifestations of disease activity, levels of disease-specific humoral autoimmunity (antinuclear antibodies (ANAs)), and assessments of the overall breadth of humoral autoimmune reactions by autoantigen microarrays.

## Subjects and methods

### Experimental subjects, samples, and clinical data

Study samples and de-identified clinical data are obtained from normal female volunteers and from female patients seen in clinics at the Penn State Milton S Hershey Medical Center. The study was approved by the Institutional Review Board of the Penn State College of Medicine and the M S Hershey Medical Center, and all subjects gave informed consent. De-identified subject samples included serum for autoantibody analyses and DNA for genotyping. Clinical data on individuals with lupus included age, number and type of lupus diagnostic criteria (26), scores for disease activity (Systemic Lupus Erythematosus Disease Activity Index (SLEDAI)) (27, 28), and medications at the time of entry into the study.

### Androgen receptor CAG repeat genotyping, *AR* gene methylation analysis, and calculation of weighted *AR* CAG repeat length

Genomic DNA was isolated from peripheral whole blood or from buffy coat cells using the QIAamp DNA Mini Kit (Qiagen) following the manufacturer's protocol. DNA was quantitated using a Nanodrop 2000c spectrophotometer.

The *AR* exon 1 CAG repeat length was measured for both alleles in each subject using a PCR-based technique as described previously (23). DNA (30 ng) was used as a template for amplification of part of the first exon of the androgen receptor gene using a FAM-labeled forward primer (5′-FAM GCT GTG AAG GTT GCT GTT CCT CAT-3′), an unlabeled reverse primer (5′-TCC AGA ATC TGT TCC AGA GCG TGC-3′), Titanium Taq DNA polymerase (Clontech), and 200 μM dNTPs. PCR was performed in an Applied Biosystems 2720 Thermal Cycler using the following cycling parameters: 94 °C for 3 min, then 35 cycles of 94 °C for 30 s, 63 °C for 20 s, and 72 °C for 30 s, and a final extension step at 72 °C for 3 min. Amplification of PCR products was confirmed by agarose gel electrophoresis. In preparation for analysis of the sizes of the PCR products from each DNA template by capillary electrophoresis, 0.5 μl of each PCR product diluted in water, 0.5 μl GeneScan 600 LIZ Size Standards v2.0 (Applied Biosystems), and 9.0 μl Hi-Di Formamide (Applied Biosystems) were mixed together in separate wells of a 96-well plate. The plate was covered with film, heated at 95 °C for 3 min, and then placed on ice. The plate was loaded and run on an Applied Biosystems 3130*xl* Genetic Analyzer in the Penn State College of Medicine Molecular Genetics Core Facility. Determination of the sizes of the PCR products was performed using the GeneMapper Software (Applied Biosystems).

Methylation analysis was carried out to detect the degree of inactivation of each *AR* allele using the CpG methylation-sensitive restriction endonucleases HpaII and HhaI (18, 29). Restriction sites for these enzymes (two for each enzyme) are within the DNA fragment (∼280 bps) amplified by the primers described above and immediately 5′ of the CAG repeat, so that methylated DNA would be uncut by either enzyme and would subsequently be amplified in the PCR described above. DNA samples of 300 ng were incubated in a 30 μl volume at 37 °C for 12 h with or without enzyme (with buffer and 100 μg/ml BSA only) for a mock digestion to produce uncut DNA. Digestion of DNA was confirmed by electrophoresis of half of each sample on a 0.8% agarose gel in TBE buffer. Aliquots of the undigested or digested DNA samples were amplified and analyzed by capillary electrophoresis as described above. Peak areas of each amplified allele were determined using the GeneMapper Software (Applied Biosystems).

The percentage of each allele that was unmethylated (active) was determined by calculating the ratio of the respective peak areas in the enzyme-digested samples. As individual alleles usually do not amplify with equal efficiency, we used a correction based on the amplification of each allele in the undigested samples (18, 30). 

A weighted mean *AR* CAG repeat length was calculated (31) from the following variables:

(a) peak area of allele 1 in digested samples
(b) peak area of allele 2 in digested samples
(c) peak area of allele 1 in undigested samples
(d) peak area of allele 2 in undigested samples
(e) number of CAG repeats in allele 1
(f) number of CAG repeats in allele 2

These variables were used in the following equations:

Fractional inactivation of allele 1:



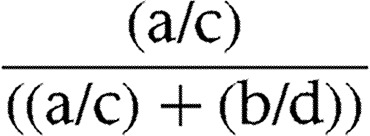



Fractional inactivation of allele 2:



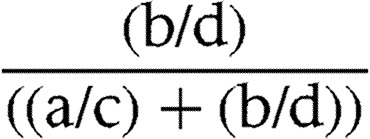



Weighted mean *AR* CAG repeat length:



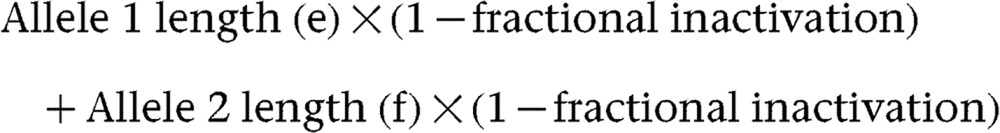



Samples were analyzed in this fashion with both HpaII and HhaI (Promega). The results were uniformly concordant and the final weighted mean *AR* CAG repeat length was the average of these two determinations.

### Antinuclear antibody assay

Levels of ANAs were measured in serum samples using the ELISA technique (Inova Diagnostics, San Diego, CA, USA). A positive result in this assay is defined as >20 ELISA units. ANA results using this technique correlate with those obtained by immunofluorescence assays (32).

### Autoantigen microarray

Individual serum samples (5 μl) were analyzed for IgG and IgM autoantibodies directed against the components of an 84-component autoantigen array described previously (23, 33). Arrays were prepared and run in the University of Texas Southwestern Medical Center Microarray Core Facility. Data were normalized for total IgG or IgM levels in each sample. Analysis of the normalized fluorescence intensities was carried out using the open-source software program Cluster 3.0 (M Eisen, Stanford University, with manual updated by M de Hoon, University of Tokyo) and the results were visualized using the open-source software program Treeview 1.6. In addition, the normalized fluorescence intensities observed for each specific antigen in the 24 normal subjects were used to define a cutoff for a positive test based on the mean+3 s.d. of the values observed. For the lupus subjects, values above this level were scored as positive tests for each respective autoantibody.

### Statistical analysis

Differences in mean *AR* CAG repeat lengths for alleles and for weighted mean *AR* CAG repeat values based on methylation analysis were determined in the study populations by a *t*-test. The Gaussian curve fitting for the frequency distribution of *AR* CAG repeat lengths was done using the GraphPad Prism 6 Software (La Jolla, CA, USA). The same software was used to determine linear correlations between individual *AR* CAG repeat lengths and measures of disease activity. Hierarchical cluster analysis of normalized microarray data was carried out using Cluster 3.0 and the results were prepared for visual presentation as heat maps using Tree View 1.6. Fisher's exact test was used to analyze autoantigen microarray data as discrete variables (positive tests vs negative tests, as described above) in individuals with short (≤19) *AR* CAG repeats compared with those with long (>19) *AR* CAG repeats.

## Results

**Figure 1:**
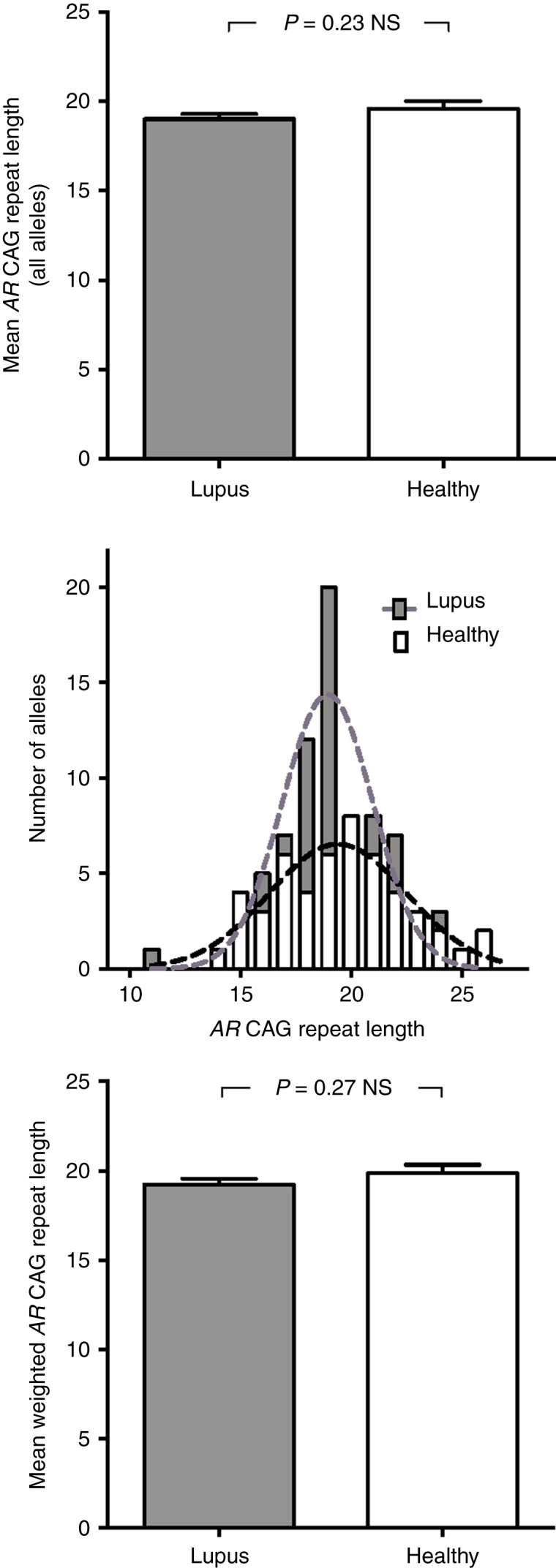
*AR* CAG repeat length does not differ between lupus subjects and healthy controls. The length of each *AR* allele CAG repeat was determined in 25 healthy female control volunteers and 39 women with lupus. Top panel: mean *AR* CAG repeat length of all alleles in each group. Middle panel: distribution of *AR* CAG repeat lengths for all alleles in healthy controls and lupus subjects. Bottom panel: mean+s.e.m. of the weighted mean *AR* CAG repeat length determined by *AR* gene methylation analysis for both populations.

To assess whether *AR* CAG repeat lengths differed between healthy individuals and subjects with lupus, we genotyped 25 healthy female volunteers and 39 women with a diagnosis of lupus who are subjects in a registry at the Milton S Hershey Medical Center. No difference was observed in the average length of the *AR* exon 1 CAG repeat in all alleles between the healthy control group and the lupus subjects (mean CAG repeat length of 19.03±0.28 for lupus subjects vs 19.69±0.47 for healthy controls; Fig. 1, top panel). *AR* CAG repeat length in both the healthy and the lupus populations appeared to be normally distributed (the Gaussian curve fit *r* ^2^=0.83 for lupus subjects and *r* ^2^ =0.86 for healthy controls; Fig. 1, middle panel).

X chromosomal inactivation could conceivably alter effective *AR* CAG repeat length and thus modulate androgen action in any given androgen target cell. We therefore analyzed allele-specific methylation in peripheral blood white cells from these healthy controls and lupus subjects. Weighted mean *AR* CAG repeat lengths were determined for each individual using methylation-sensitive restriction digests to identify inactivated alleles. We found no significant difference in the weighted mean *AR* exon 1 CAG repeat length between lupus subjects and healthy controls (19.24±0.33 for lupus subjects vs 19.86±0.47 for healthy controls; Fig. 1, bottom panel). These data are all consistent with the formulation that inherited differences in *AR* CAG repeat length (and, consequently, androgen sensitivity) do not play any primary role in the predisposition to the development of autoimmunity in individuals with lupus.

Nonrandom patterns of X chromosomal inactivation (‘skewing’) have been reported to occur more frequently in individuals with autoimmune disease (34). We analyzed each individual subject's X chromosomal methylation at CpG islands in close proximity to exon 1 of the androgen receptor gene to assess whether preferential expression of *AR* alleles of greater or lesser length might be more prevalent in the lupus subjects. Skewed X inactivation can be defined by a variety of criteria – usually ranging from >70 to >90% inactivation (methylation) of one allele. We noted such skewed methylation in 7.8–32.8% of individuals (Table 1) based on the different defining criterion used. In none of these instances was a difference in X inactivation pattern demonstrable between lupus subjects and the healthy controls.

While there was little reason to expect that the genetic determinants of androgen insensitivity could influence overall susceptibility to autoimmune disease, we hypothesized that inherited variation in sensitivity to hormone action might affect aspects of the course, severity, or breadth of autoimmune reactivity. We examined in detail a subset of individuals (eight healthy controls and 23 lupus subjects for whom we had available clinical data and serum samples) to test whether the *AR* CAG repeat length might be related to differences in clinical features of lupus or in the manifestations of humoral autoimmunity that characterize the disorder. Of the 23 subjects with lupus, 13 had been treated with hydroxychloroquine, nine had received treatment with a pharmacological dose of glucocorticoids, and two had received mycophenolate mofetil. There was no difference in the weighted mean *AR* CAG repeat length among the groups who had received different therapies, and none of them differed from the mean for the total subject population.

**Figure 2:**
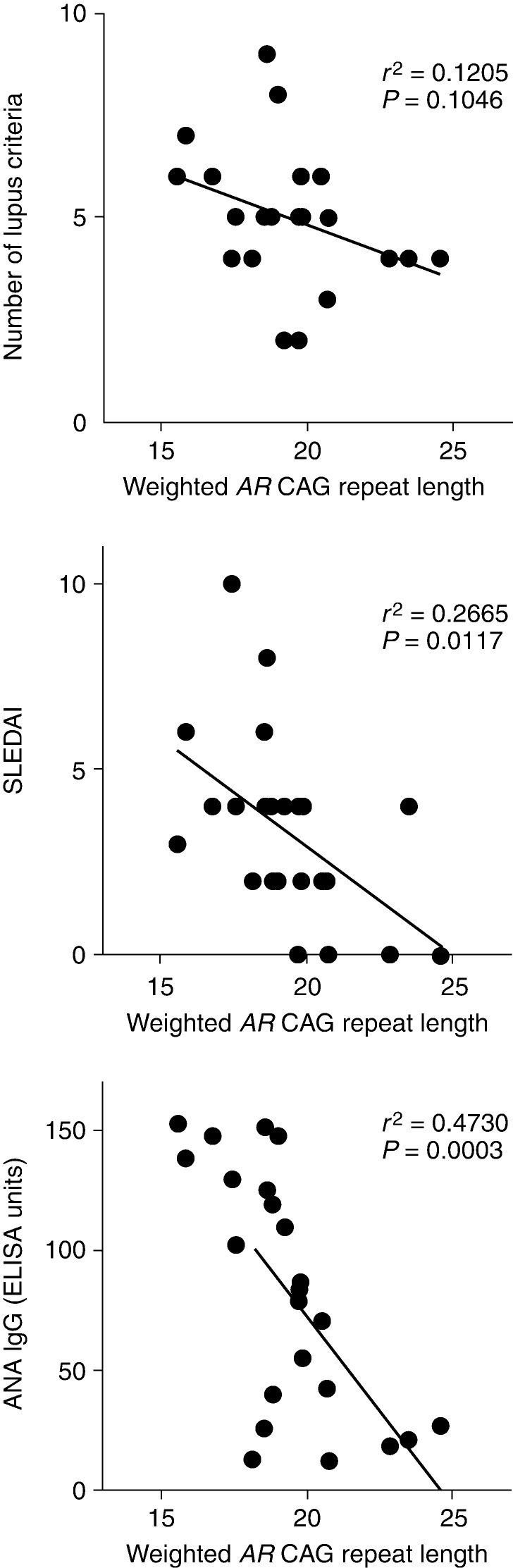
Weighted mean *AR* CAG repeat length is inversely correlated with clinical and humoral immune manifestations of lupus at the time of entry into the study. Top Panel: number of lupus diagnostic criteria identified in patients as a function of the weighted mean *AR* CAG repeat length. Middle panel: Systemic Lupus Erythematosus Disease Activity Index as a function of the weighted mean *AR* CAG repeat length for 23 lupus subjects. Bottom panel: levels of anti-nuclear antibodies (ANAs) determined by immunoassay are shown as a function of the weighted mean *AR* CAG repeat length.

The clinical criteria used to diagnose lupus and a clinical phenotyping score (SLEDAI) used to assess disease activity both showed evidence of an inverse correlation with the weighted mean *AR* CAG repeat length in the lupus subjects. The number of lupus diagnostic criteria observed for each patient at the time of entry into the registry failed to show a linear correlation with the *AR* CAG repeat length when analyzed as a continuous variable (*r* ^2^ =0.1205; *P*=0.1046). The SLEDAI showed a significant inverse correlation with the weighted mean *AR* CAG repeat length (*r* ^2^ =0.2665; *P*=0.0117; Fig. 2, middle panel).

The characteristic immunological aberration in SLE involves humoral autoimmunity, and autoreactivity against nuclear components, including DNA, is a hallmark of the disease. We found a significant inverse correlation between the level of expression of antinuclear antibodies and the weighted mean *AR* CAG repeat length in our cohort of lupus subjects (*r* ^2^ =0.4730; *P*=0.0003; Fig. 2, bottom panel).

**Figure 3:**
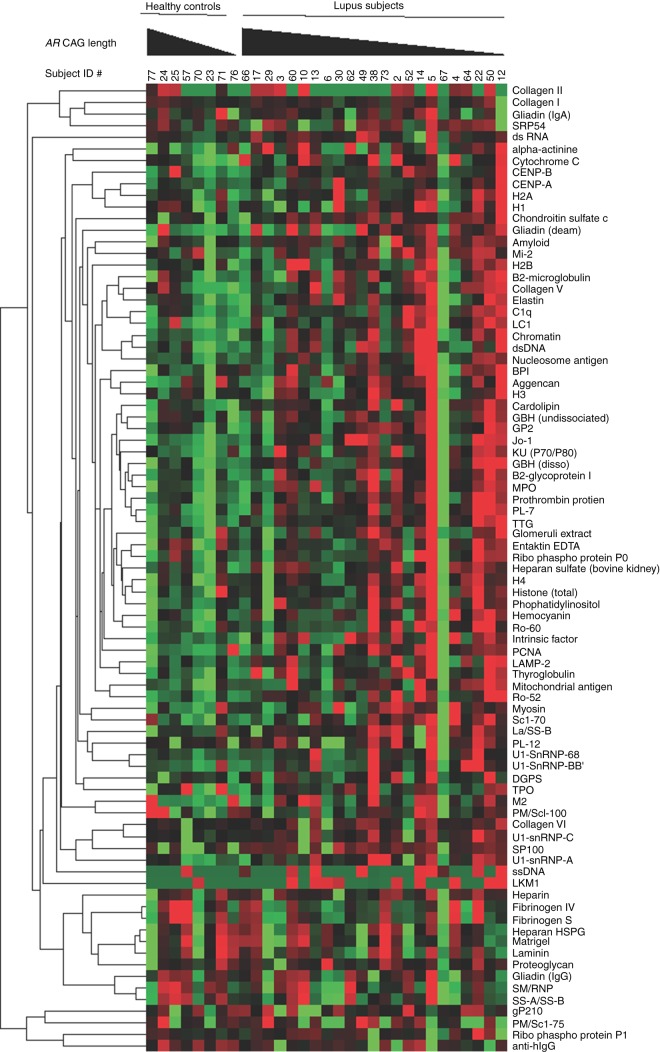
Expression of IgG autoantibodies is greater in female lupus subjects with a short weighted mean *AR* CAG repeat length. Sera from eight healthy control women and 23 women with lupus were analyzed for expression of IgG autoantibodies using a protein microarray technique. Each column represents an individual subject, grouped as healthy controls and lupus subjects, and arranged in the order of decreasing weighted mean *AR* CAG repeat length. Each row represents antibody reactivity against a single autoantigen. Hierarchical clustering of normalized data was used to create the heat map. More intense red indicates reactivity greater than the mean of the group and more intense green indicates reactivity below the mean.

**Figure 4:**
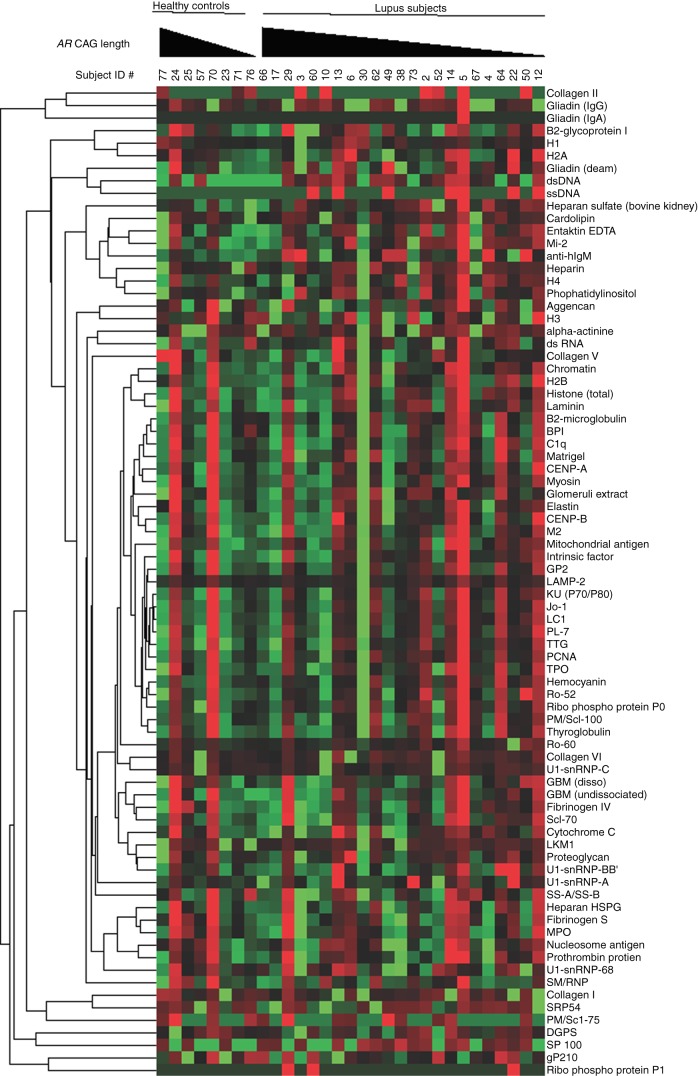
Expression of IgM autoantibodies is greater in female lupus subjects with a short weighted mean *AR* CAG repeat length. Sera from eight healthy control women and 23 women with lupus were analyzed for expression of IgM autoantibodies using a protein microarray technique. Each column represents an individual subject, grouped as healthy controls and lupus subjects, and arranged in the order of decreasing weighted mean *AR* CAG repeat length. Each row represents antibody reactivity against a single autoantigen. Hierarchical clustering of normalized data was used to create the heat map. More intense red indicates reactivity greater than the mean of the group and more intense green indicates reactivity below the mean.

**Figure 5:**
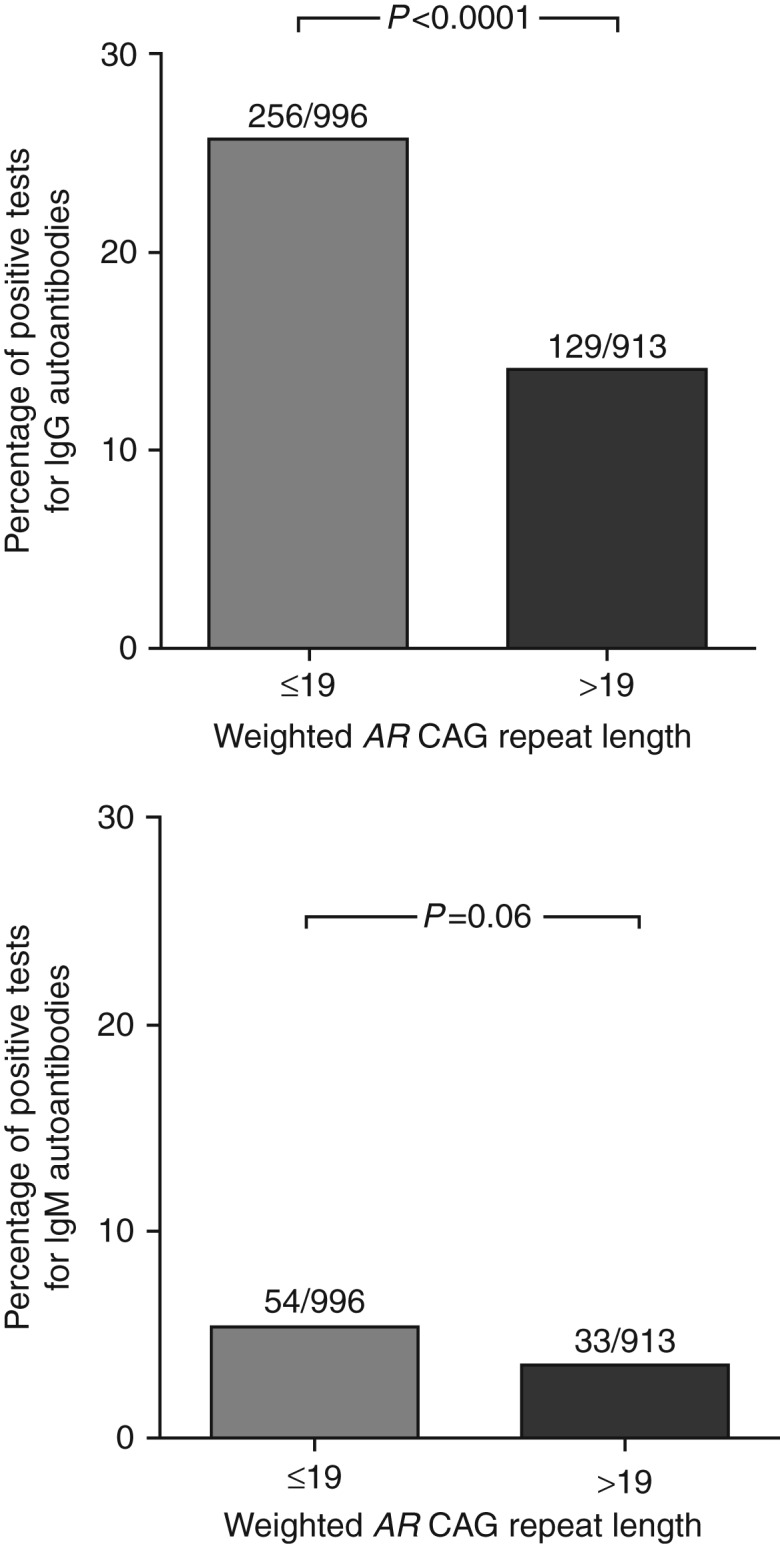
IgG but not IgM autoantibody expression is quantitatively greater in female lupus subjects with a short weighted mean *AR* CAG repeat length. The mean±3 s.d. of the mean fluorescence intensity of normal subjects for each autoantigen specificity was calculated as the threshold for a positive test. The number of positive tests for female lupus subjects with a weighted mean *AR* CAG repeat length of ≤19 or >19 was determined for each autoantibody specificity. Top panel, IgG autoantibodies. Bottom panel, IgM autoantibodies. Data were analyzed by Fisher's exact test.

Humoral autoimmunity in lupus extends to loss of tolerance for a wide variety of autoantigens, and we investigated whether the *AR* CAG repeat length might be related to differences in the breadth of the autoimmune response as well. We used an established autoantigen array technique to correlate patterns of autoantibody expression with the length of the weighted mean *AR* CAG repeats in female subjects with lupus. Cluster analysis of the specific patterns of IgG autoantibody reactivity revealed qualitative evidence of more exuberant autoreactivity in individuals with a shorter *AR* CAG repeat length (Fig. 3). Individual lupus patients with shorter *AR* CAG repeat lengths showed more intense IgG reactivities (red color) against individual antigens and showed more numerous autoantibody specificities than did the lupus subjects with longer *AR* CAG repeat lengths. A similar (although less pronounced) qualitative trend appeared for IgM autoantibodies (Fig. 4). To assess these parameters more quantitatively, we analyzed the microarray data by examining each individual's autoantibody reactivities in comparison to the average signal observed from the serum of the healthy controls. Using such positive/negative criteria, we found 385 IgG tests to be positive out of 1909 (20.1%) and 87 IgM tests to be positive out of 1909 (4.5%). Analysis using the weighted *AR* CAG length as a dichotomous variable (≤19 vs >19) revealed a statistically significant increase in the number of autoreactive IgG antibodies in individuals with a shorter *AR* CAG length (*P*<0.0001 for IgG; Fig. 5, top panel). The frequency of positive IgM autoantibody tests was not significantly different between individuals with shorter and longer weighted mean *AR* CAG repeat lengths (Fig. 5, bottom panel).

## Discussion

Since the time of Talal's seminal experiments in the NZB/W mouse, the preponderance of evidence has been that androgenic signaling in the immune system was generally suppressive (1, 2, 3). Our prior studies in men with lupus supported the formulation that attenuated androgen signaling through long *AR* CAG repeats resulted in increased manifestations of autoimmune phenomena (23). The studies described here revealed an opposite result – that, in women with lupus, such genetic variants of the androgen receptor gene (long *AR* exon 1 CAG repeat lengths) that are generally believed to result in diminished efficiency of hormonal signaling are associated with decreased severity of clinical and serological parameters of autoimmunity. Conversely, females with lupus who had shorter *AR* exon 1 CAG repeats (which are associated with relatively amplified hormonal action) were found to have greater disease activity and more robust humoral autoimmune responses. Our current data are also similar to the observations in women with rheumatoid arthritis – in whom a shorter *AR* CAG repeat length was noted to be associated with earlier onset and more aggressive disease course (24) – and to a recent study of women with SLE in which the *AR* CAG repeat length was inversely correlated with the SLICC/ACR index of disease damage (25). Differences in androgen sensitivity conferred by *AR* CAG repeat variation in women do not appear to be compensated by alterations in circulating hormone levels. While longer *AR* CAG repeats are associated with higher levels of circulating androgens in men (35, 36), women with longer *AR* CAG repeats have previously been found to have lower testosterone levels (at least during the follicular phase of the menstrual cycle) (37).

Some limitations of the present study might be noted. First, a relatively small number of subjects have been evaluated, and the possibility of some unrecognized unique characteristics of our study population cannot be excluded. Our subjects exhibited normal distribution of their *AR* allele CAG repeat lengths and their enrollment in the Hershey Medical Center lupus registry was not biased for any particular manifestation of disease, for severity of manifestations, or for specific therapy. The study is limited by its cross-sectional design. Our clinical data (diagnostic criteria and SLEDAI score) were based on disease manifestations present at the time of each subject's enrollment in the study and further longitudinal data on the relationship of *AR* genotype to the course of disease would certainly be of interest. Finally, our data are from DNA samples derived from whole blood samples or buffy coat cells. Obviously, while this would not affect the genotyping of the individual *AR* alleles, the presence of different patterns of X chromosomal inactivation in different cell types could affect the calculation of the weighted mean *AR* repeat length. It might be argued that specific subsets of B cells at different points during the lymphopoiesis pathway or even bone marrow stromal cells (which have been found to mediate androgen signals on B cell development in marrow) (38) would potentially be more relevant cells for study.

Our current studies do not explain how attenuation of androgen action could result in amelioration of features of the autoimmune phenotype in women with lupus. It is possible that X chromosomal genes in linkage disequilibrium with *AR* are, in fact, responsible for the observed effects, and several candidate genes on the X chromosome have been identified as susceptibility factors for the development of lupus. Interleukin 1 receptor-associated kinase 1 (*IRAK1*; located at Xq28) and toll-like receptor 7 (*TLR7*; located at Xp22.2) are both such X chromosomal genes for which both human association studies and mechanistic experiments in mice support roles in lupus pathogenesis (39, 40), but their distance from the *AR* gene (at Xq12) would certainly seem to preclude such linkage disequilibrium. A number of other X chromosomal genes, including that encoding CD40 ligand, have been found to be overexpressed in T cells of women with lupus as a consequence of gene demethylation on an otherwise inactivated X chromosome (41). Some of these genes, including *CXCR3*, *OGT*, and *MIR421* (on X q13), are in closer proximity to the *AR* gene.

A second possibility is that inherited differences in androgen sensitivity might alter the steroid hormonal milieu of an individual by altering feedback regulation of gonadotropins or other mechanisms. In men with long *AR* CAG repeats, both estrogen and androgen levels are increased (36), and it has been argued that these parallel increases in both hormones in the face of attenuated androgen action result in a net increase in the estrogen:androgen bioactivity (36). Women with longer *AR* CAG repeat lengths have been reported to have lower circulating androgen levels (37). Whether any other changes in the steroid metabolome (such as reduction in effective estradiol levels at immune cell targets) might occur in females with diminishing androgen sensitivity and how they might affect immune function are not known.

It is also possible that interactions between an individual and the environment might account for the relationship between inherited androgen sensitivity and the manifestations of autoimmunity. The microbiotic environment of the *Nod1* mouse has now been shown to interact with the animal's hormonal milieu – with consequent impact on the expression of autoimmunity (42, 43, 44, 45). Whether inherited variation in androgen sensitivity influences the establishment of the specific composition of the human microbiome, and whether the microbiome interacts with the hormonal milieu and with genetically established levels of hormone sensitivity to influence immune function are topics that are essentially unexplored.

A variety of mechanisms have been proposed to account for the remarkable sexual dimorphism of most human autoimmune diseases (46). The effects of X chromosomal genes (47, 48, 49), the effects of Y chromosomal genes (50, 51, 52, 53), fetal microchimerism in mothers (54), the action of gonadal steroid hormones (55), and the influence of endogenous microbial populations that differ between males and females (42, 43) have all been suspected to influence the expression of autoimmune phenomena (56). Our findings described in this work challenge our simple view that increased capacity for the transmission of androgenic hormonal signals would be relatively immunosuppressive. The complex interactions of genes, hormones, hormone receptors, and the environment need to be considered in examining the influence of each of these parameters on the expression of autoimmunity.

## Figures and Tables

**Table 1:** Skewing of X chromosomal methylation at *AR* locus in healthy controls and lupus subjects.

**Criterion** (% inactivation of one allele)	**Percentage of total sample skewed**	**Percentage of healthy controls skewed**	**Percentage of lupus subjects skewed**	**Difference between healthy and lupus subjects** (*P* value by Fisher's test)
>70%	32.8	24	38.4	0.2823 (NS)
>75%	23.4	16	28.2	0.3676 (NS)
>80%	18.7	16	20.5	0.7512 (NS)
>90%	7.8	8	7.6	1.000 (NS)
